# Ridge Preservation Procedures after Tooth Extractions: A Systematic Review

**DOI:** 10.1155/2018/8546568

**Published:** 2018-07-03

**Authors:** Gabriella Balli, Andreas Ioannou, Charles A. Powell, Nikola Angelov, Georgios E. Romanos, Nikolaos Soldatos

**Affiliations:** ^1^Department of Periodontics and Dental Hygiene, School of Dentistry, University of Texas Health Science Center at Houston, Houston, TX, USA; ^2^Department of Surgical Dentistry, Division of Periodontics, School of Dental Medicine, University of Colorado, Anschutz Medical Campus, Aurora, CO, USA; ^3^Department of Periodontology, Stony Brook University School of Dental Medicine, Stony Brook, NY, USA; ^4^Department of Oral Surgery and Implant Dentistry, Johann Wolfgang Goethe University of Frankfurt, Frankfurt, Germany

## Abstract

**Background:**

The purpose of this systematic review was to accurately assess the procedural success of ridge preservation technique through the application of strict inclusion and exclusion criteria.

**Data Sources:**

A methodical search of PubMed of the US National Library of Medicine and the Cochrane Central Register of Controlled Trials was conducted for applicable articles. Only randomized controlled trials comparing ridge preservation treatment with a nongrafting control, ten-subject minimum sample size, and three or more months of follow-up were included in our study.

**Types of Studies Reviewed:**

In a screening between January 1980 and September 2017, articles meeting predetermined criteria were further examined in a qualitative data analysis. A thorough search of the databases provided 1876 articles. Of these records, 174 were assessed for eligibility through the systematic employment of inclusion and exclusion criteria.

**Results:**

Two records were appropriate for further data analysis. One study used a mixture of a deproteinized cancellous bovine bone and porcine collagen fibers in a block form (DBB/CF), while the other study used leukocyte-platelet-rich fibrin (L-PRF). The use of DBB/CF reduced the magnitude of vertical bone resorption, yet the study showed high risk of bias. The use of L-PRF reduced the magnitude of both the horizontal and vertical crestal bone resorption; however, the low sample size created wide standard deviations between the test and control groups. Inherent weaknesses were present in both studies. Through methodical analysis of both records, the dissimilarities prevented the conduction of a meta-analysis.

**Implications of Key Findings:**

Within the limitations of this systematic review, L-PRF reduced the magnitude of vertical and horizontal bone resorption, which places L-PRF as a potential material of choice for ridge preservation procedures.

**Conclusions:**

Within the limitations and weaknesses of both studies, the use of DBB/CF prevented the vertical crestal bone resorption while the L-PRF prevented both the horizontal and vertical crestal bone resorption. More randomized controlled clinical trials are needed to eliminate all the confounding factors, which bias the outcome of ridge preservation techniques.

## 1. Introduction

With the advent of implant dentistry, there has been an increased emphasis placed on preserving and maintaining the implant-bearing environment. Disruption to the oral environment by tooth extraction can compromise the integrated tissue morphology, inducing healing mechanisms similar to those of new tissue formation [1]. Two very important components for the socket integrity (bundle bone and periodontal ligament fibers) vanish by the 14th day after extraction [1]. Schropp et al. determined that major changes of an extraction site are occurring within 12 months, during which time a 50% (5–7 mm) reduction of alveolar ridge width can be observed, with two-thirds of this reduction occurring within the first three months [2]. These changes at the extraction site may be due to the loss of periodontal ligament fibers, bundle bone, and loss of blood supply. These morphologic changes pose significant challenges in restorative treatment, as soft tissue recession and buccal plate resorption define the anatomical profile of the socket and may narrow the viable treatment options [3]. Elian et al. [3] have classified sockets following extraction into three simplified socket types: Type I sockets, intact with normal levels of facial soft tissue and buccal plate bone, provide anatomical predictability with implant placement leading to esthetically satisfying results; Type II sockets with normal facial soft tissue appearance yet partially missing buccal plate bone creates the greatest challenge in proper diagnosis; and Type III sockets exhibit reduced facial soft tissue and buccal plate bone, thus requiring guided bone regeneration to reconstruct insufficient tissues. Repairing socket defects to create an anticipated foundation for implant placement is the rationale behind the surgical technique of ridge preservation [3]. According to the American Academy of Periodontology Glossary of Periodontal Terms, ridge preservation is a surgical procedure aimed at preventing ridge collapse and preserving ridge dimension after tooth extraction, typically done for purposes of implant site development [4].

Allograft, xenograft, and alloplastic materials, along with the autogenous bone, have been utilized in preserving the alveolar ridge to maximize implant outcomes [5]. During postextraction healing, vertical and horizontal bone loss is expected, yet the use of grafting materials can provide dimensional stability to the alveolar ridge [6, 7].

In a study performed by Araújo et al., deproteinized cancellous bovine bone and porcine collagen fibers in a block form (DBB/CF) placed in fresh extraction sites proved to be effective in offsetting hard tissue reduction with approximately 3% reduction in the cross-sectional area of the grafted alveolar ridges, as compared to nongrafted sites with roughly 25% of reduction [8]. Araújo and Lindhe differentiated the xenograft bone from the autologous bone, as the autologous bone was unsuccessful in the formation of a new bone and the prevention of ridge resorption after extraction [9]. Cardaropoli et al. compared an extraction control to sockets grafted with a DBB/CF. The DBB/CF provided horizontal ridge width loss of 0.71 mm with ridge preservation, compared to 4.04 mm width loss with spontaneous healing, irrespective of the buccal bone thickness [7].

In a study performed by Iasella et al., the use of mineralized freeze-dried bone allograft (FDBA) resulted in a 1.2 ± 0.9 mm loss of horizontal ridge width, when compared to a control, untreated extraction socket (2.6 ± 2.3 mm). In addition, this study showed a gain of vertical ridge height of 1.30 ± 2.00 mm in the FDBA group indicating that ridge preservation has the capacity to provide necessary bone volume for implant placement [6]. To augment the bone grafting process through space maintenance and preventing migration of surrounding soft tissue into the socket, resorbable and nonresorbable membranes are used [10, 11]. Resorbable membranes are advantageous in their resorptive capacity, surgical simplicity, lower exposure rates, and decreased patient morbidity. However, these membranes can compromise the healing environment with their variable resorption rates, need for tenting screws to prevent collapse, incomplete resorption, associated material memory, and potential movement amplified by the membrane microenvironment [10]. The most common resorbable membrane used is a collagen membrane, designed to match the properties of the periodontal connective tissues [10, 11]. These membranes act as a scaffold to amplify tissue flap thickness, promoting primary wound closure by chemotaxis of periodontal ligament and gingival fibroblasts, and encourage wound healing through hemostasis and maintenance of membrane integrity [11]. Prolonged resorption rates, linearly related to the degree of cross-linking, adequately prevent apical migration of the epithelium as the membrane remains intact during epithelial proliferation [11].

Various ridge preservation techniques are presented in the literature. Amongst these methods, the “ice cream cone” technique has been acceptable for cases of buccal plate defects (Type I and II sockets), after extraction [3, 12]. As confirmed by Tan-Chu et al., the “ice cream cone” technique performed with bone allograft and resorbable collagen membrane restored the buccal plate with a small ridge width reduction of 1.32 mm, proving the success of this procedure [12]. Jiang et al. suggested a pressure-bearing microtitanium stent to preserve the contour of the extraction socket [13]. The microtitanium stent, even with its distortion, is acceptable in preserving the alveolar ridge, yet the stent fails to act as a barrier membrane [13]. SocketKAP™ and SocketKAGE™ prefabricated devices have also been studied for their effectiveness in ridge preservation [14, 15].

Local and systemic patient factors can compromise the definitive outcome. Smoking has been associated with dimensional reduction of postextraction sockets, where a 0.5 mm bone loss is expected in smokers as compared to nonsmokers [16]. As ridge preservation continues to evolve, there is an increased demand for appropriate assessment of procedural success, which is difficult without omission of all possible confounding factors.

This systematic review aims at eliminating these confounding factors through the development of criteria based on the study design, method of obtaining study results, number of subjects enrolled per study, and systemic factors of subjects. In addition, this systematic review aims at answering the focus question, If extraction and ridge preservation with the use of biologic materials can influence the magnitude of horizontal and vertical bone resorption, compared to spontaneous healing in patients needing extraction and ridge preservation?

## 2. Materials and Methods

### 2.1. Search Strategy

Our methodology followed the materials and methods of a previous systematic review from Kotsakis et al. [17]. The PRISMA (Preferred Reporting Items for Systematic Reviews and Meta-Analyses) guidelines for reporting a systematic review were followed [18]. Two electronic databases, PubMed of the US National Library of Medicine and the Cochrane Central Register of Controlled Trials, were methodically searched for applicable articles, in the English language.

Selected key words “*ridge preservation*,” “*alveolar ridge preservation*,” and “*socket preservation*” were applied, and results were individually screened in a follow-up equal or greater to three months, between January 1980 and September 2017.

In addition to the electronic search, manual searching of selected journal titles was performed: *Journal of Periodontology*, *Journal of American Dental Association*, *Journal of Clinical Periodontology*, *The International Journal of Oral and Maxillofacial Implants*, *The International Journal of Periodontics and Restorative Dentistry*, *Clinical Implant Dentistry and Related Research*, *Clinical Oral Implants Research*, *and Implant Dentistry*.

At the first phase of selection, the titles and abstracts of all articles found through the electronic and manual searches were screened, independently, by two reviewers (Gabriella Balli and Nikolaos Soldatos). When studies met the inclusion criteria or when data from the abstracts were insufficient to determine eligibility, the full article was obtained. Following the initial phase of selection, the two reviewers scrutinized the full text articles of all relevant studies for final inclusion. If there was disagreement between the two reviewers, consensus was achieved by discussion with a third reviewer (Andreas Ioannou).

### 2.2. PICOS

The criteria for inclusion of studies for this review were organized by the PICOS (Population, Intervention, Control, Outcome, Setting, and Study design) approach as follows: Population—subjects in the included trials must have been humans undergoing the ridge preservation procedure; Intervention—the intervention of interest was ridge preservation; Control—randomized control studies; Outcome—dimensional changes of the ridge on the site of interest with the help of the CBCT was set as the primary outcome variable; Setting—university, hospital, and private practice; and Study designs—randomized control clinical trials.

### 2.3. Eligibility Criteria

Studies were filtered to include randomized controlled trials, comparing ridge preservation treatment with a nongrafting control, ten-subject minimum sample size, and three or more months of follow-up. Cone beam computed tomography (CBCT) was used to obtain study results, before and after rendered treatment. The population of interest was narrowed to healthy human subjects aged 18 years and older.

## 3. Results

**Figure 1:**
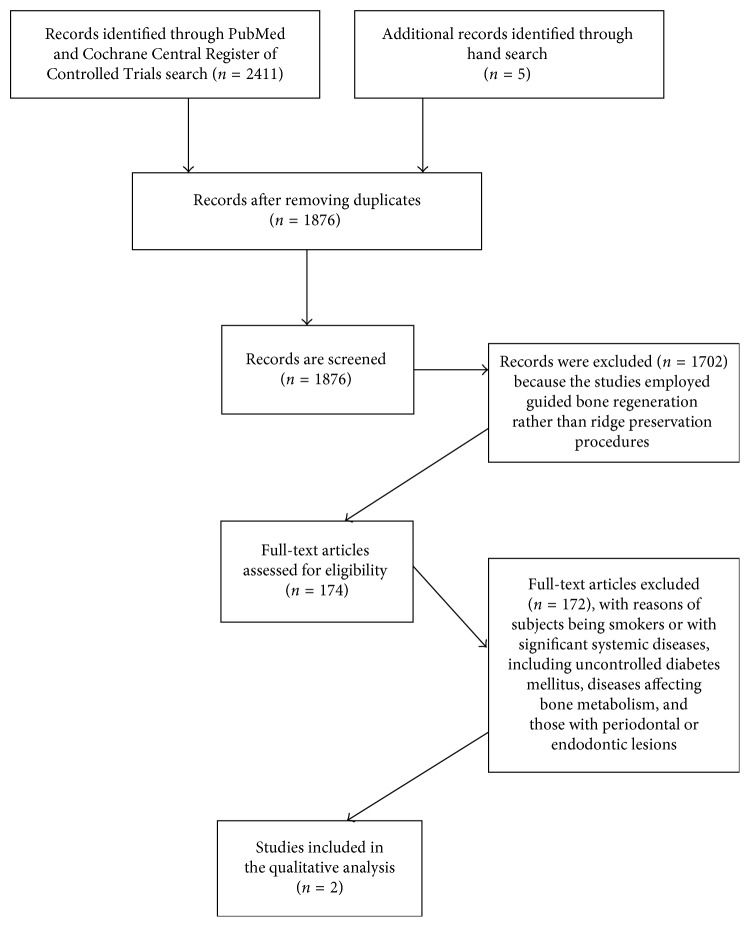
The PRISMA flow diagram.

A PRISMA flow diagram was developed to display the search results (Figure 1) [18]. The literature search of both databases provided a total of 2,411 studies. Through the manual search, five supplementary studies were added. After the duplicates were removed, 1,876 papers were available for screening. Of the 1,876 articles retrieved, 1702 articles were excluded because they employed guided bone regeneration techniques rather than ridge preservation. Full-text articles were obtained for the 174 remaining articles. One hundred and seventy-two studies were excluded because the subjects were smokers or with significant systemic diseases, including uncontrolled diabetes mellitus, diseases affecting bone metabolism, and those with periodontal or endodontic lesions. Two articles met the inclusion criteria for qualitative data analysis.

The studies included in the final review are displayed in Table 1 [19, 20]. Pang et al. included sixty patients in a randomized controlled clinical study. The authors performed ridge preservation with a mixture of a DBB/CF and a collagen membrane. The flap management involved two vertical releasing incisions on the mesial and distal areas, respectively. The test group was divided on two buccal defect levels: level A (defects between 3 and 5 mm from the crest) and level B (defects 5 mm or more). The control group consisted of spontaneous healing sockets. A CBCT was used preoperatively and postoperatively to measure the bone levels between the groups. The patients were followed up for 6 months postoperatively. Regarding the changes in the vertical dimension, both level A and level B test groups had statistically significant less bone loss compared to the control groups (*level A*: test, −1.53 ± 0.26 mm; control, −2.92 ± 0.31 mm) (*level B*: test, −2.48 ± 0.22 mm; control, −3.17 ± 0.37 mm). The horizontal dimensions did not show significant differences over the control and test groups in both the levels (*level A*: test, −2.87 ± 0.25 mm; control, −3.26 ± 0.44 mm) (*level B*: test, −3.05 ± 0.18 mm; control, −3.82 ± 0.33 mm). In both the groups, the implants were placed and measured through the ISQ system. Three months postoperatively, the ISQ numbers did not differ significantly between the groups [19].

Temmerman et al. included twenty-two patients in a randomized controlled clinical study. Two to three leukocyte-platelet-rich fibrins (L-PRF) were placed in the sockets of the test groups. The control groups were left to heal spontaneously. The patients were followed up for 3 months postoperatively. The test and control groups were divided to horizontal and vertical resorption sites as well as buccal and lingual surfaces. Moreover, the horizontal groups were subdivided to 3 more groups: 1, 2, and 5 mm below the crest (HW-1, HW-3, and HW-5). The buccal surface of the horizontal dimension showed statistically significant bone resorption at the control group compared to the test group, at HW-1 and HW-3 levels (*HW-1*: control, −3.3 ± 2.6 mm; test, −1.2 ± 2.6 mm) (*HW-3*: control, −1.0 ± 1.1 mm; test, −0.8 ± 0.9 mm). The lingual surface of the horizontal dimension showed statistically significant bone resorption at the control group, compared to the test group only at the HW-1 level (*HW-1*: control, −2.0 ± 2.6 mm; test, −0.3 ± 1.9 mm). The buccal aspect of the vertical dimension presented statistically significant bone resorption on the control group, compared to the test group (control: −1.6 ± 1.2 mm; test: −0.1 ± 1.6 mm) [20].

Both the studies were evaluated for risk of bias based on van Tulder et al., Boutron et al., and the Cochrane Handbook of Systematic Reviews of Interventions, Chapter 8 (version 5.1, updated on March 2011) [21, 22]. Pang et al. showed higher risk of bias (Table 2) [19].

## 4. Discussion

When screening for applicable articles, this systematic review limited the population of interest by age, health, and lifestyle factors. In former systematic reviews, many of the appropriate articles contained subjects with a smoking habit [16, 23]. A systematic review published in 2009, by Van der Weijden et al., highlights one limitation of their review as the heterogeneity amongst the selected studies, stemming from differences in evaluation parameters, reasons for extraction, study designs, and study populations, amongst others. This systematic review failed to control the smoking habit amongst the study populations, which is a known confounding variable on postextraction healing, as the authors noted [16]. However, our systematic review strictly excluded studies, which included individuals with a smoking habit. This exclusion criterion functioned to eliminate the effects of smoking on the dimensional stability of the extraction socket.

In addition to confounding population variables, many studies in selected systematic reviews were not segregated by socket location, reasons for extraction, or measurement approach [5, 23]. Measurement data gathered from acrylic stents, titanium pins, and cast models, along with the use of assorted surgical protocols, proved to be inconsistent and unreliable. Similar to the systematic review by Van der Weijden et al., a 2012 systematic review published by Vignoletti et al. also included studies with subjects possessing a smoking habit along with a lack of outcome measurement standardization. In the Vignoletti et al. study, the array of measurement methods resulted in the need for subgroup analysis in order to determine the effect of measurement methods on study outcomes [23]. Juxtaposing the methodology of Vignoletti et al., our systematic review accounts for the bias induced by variable measurement methods by standardizing the evaluation of study outcomes by the inclusion of the CBCT. CBCT was introduced in the 1990s and was used to display a three-dimensional image of the jaws to identify anatomical structures such as the maxillary sinus, the inferior alveolar nerve, and the length and the resorption of roots [24]. CBCT has shown to be more accurate compared to the conventional two-dimensional radiographic methods like periapical and panoramic X-rays [25–27]. The average amount of distortion expressed as percentages are 14% for periapical X-rays, 23.5% for panoramic X-rays, and 1.8% for the CBCT's [25]. However, the problem of the CBCT is the radiation, which is higher compared to the conventional radiographic methods [24]. Significant dose reduction can be achieved by reducing the field of view to the area of interest [25].

Published in 2015, a systematic review by Jambhekar et al. contained similar limitations to other systematic reviews as measurement methods, and anatomic socket location was not consisted between the selected studies. In addition to these limitations, 6 of the 32 selected studies utilized allograft for socket preservation, which can induce bias, as this grafting material was unavailable in certain areas outside of the United States due to legal restrictions of the cadaver bone use, as discussed by the authors as well [5]. In our study, we did not exclude any type of biomaterial. The strict inclusion and exclusion criteria created a framework for this systematic review, which assisted in reducing the intrinsic bias and confounding variables that can mask the outcome of the ridge preservation technique. The studies utilized in this systematic review were filtered to include randomized controlled clinical trials, comparing ridge preservation treatment with a nongrafting control, ten-subject minimum sample size, three or more months of follow-up, and the use of CBCT as the method of evaluation. Our methodology allowed us to reduce effects of broad study designs and heterogeneous evaluation techniques.

Two studies were included for the final review [19, 20]. Pang et al. divided equally 60 patients into 2 groups; level A group was defined as having a buccal bone defect between 3 and 5 mm, while level B group as more than 5 mm. The use of DBB/CF was advantageous to spontaneous healing only for reducing the magnitude of vertical crestal resorption. The weaknesses of this study are the lack of power analysis, the lack of histology, the use of vertical releasing incisions in the test group, and the inclusion of defects more than 5 mm, since they require guided bone regeneration, rather than ridge preservation [19]. Temmerman et al. used L-PRF as a socket filling material in the test group and spontaneous healing in the control group. Horizontal and vertical bone resorption on both buccal and lingual aspects was evaluated with the use of CBCT, preoperatively and 3 months postoperatively. The horizontal bone resorption was measured in three different levels below the crest (HW-1, HW-3, and HW-5). The use of L-PRF was advantageous to spontaneous healing as far as reducing the magnitude of horizontal bone loss, both in lingual and buccal dimensions at the HW-1 level and the vertical bone loss on the buccal aspect. Although the results at the HW-1 level show statistical significance, the sample size is low, ending up in very wide standard deviations between the test and control groups. Weaknesses of this study are the lack of histology, the unequal distribution of sites between the maxilla and mandible, and the small sample size [20]. Procedure complications were noted in both studies. Pang et al. reported three sites which regenerated bone, not sufficient for implant placement, while Temmerman et al. demonstrated two sites from the control group that had to be retreated [19, 20].

Meta-analysis is typically utilized to combine results for further projection of evidence favoring the use of ridge preservation. A significant weakness in this type of statistical analysis is the source of heterogeneity amid the selected studies [16]. The weighted mean difference (WMD) should be interpreted carefully as clinical discrepancies within the selected studies may collude the statistic. Vignoletti et al. ran a meta-analysis, coupled with subgroup analysis and metaregression in attempts to assess moderator variable influence. However, this meta-analysis was unable to draw conclusions concerning the implant-related outcomes due to inadequate data [23]. In the present systematic review, authors were unable to conduct a meta-analysis as the two selected studies were dissimilar and were deemed unsuitable for combined extension of the data. The results obtained by statistical analysis in selected systematic reviews acknowledged a lack of evidence as to which biomaterials and surgical procedures should be deemed the “gold standard” in ridge preservation.

## 5. Conclusions

Within the limitations and weaknesses of both studies, the use of DBB/CF reduced the magnitude of vertical bone resorption, while the L-PRF reduced the magnitude in both the horizontal and vertical crestal bone resorption. More randomized controlled clinical trials are needed to eliminate all confounding factors, which bias the outcome of ridge preservation techniques.

## Figures and Tables

**Table 1:** The studies included in the final review.

Study	Pang et al. [19]	Temmerman et al. [20]
Methods	Randomized controlled clinical trial	Randomized controlled clinical split mouth trial

Participants	60 patients	22 patients

Surgical considerations	Extraction with two vertical releasing incisions (mesial/distal) plus collagen membrane with DBB collagen sponge	Extraction plus 2-3 PRF

Intervention	*Test group*: deproteinized collagen bovine bone covered with collagen membrane	*Test group*: leukocyte-platelet-rich fibrin
	*Control group*: spontaneous healing	*Control group*: spontaneous healing
	*Level A buccal defects*: between 3 and 5 mm	
	*Level B buccal defects*: 5 mm or more	

Measurement method	CBCT/ISQ	CBCT

Follow-up	6 months postoperatively	3 months postoperatively

Outcome	*Vertical resorption*:	*Vertical resorption (lingual)*:
	(i) Level A (*control group*): −2.92 ± 0.31 mm	−0.7 ± 0.8 mm (control group)
	(ii) Level A (*test group*): −1.53 ± 0.26 mm	−0.3 ± 1.2 mm (L-PRF group)
	(iii) Level B (*control group*): −3.17 ± 0.37 mm	*Vertical resorption (buccal)*:
	(iv) Level B (*test group*): −2.48 ± 0.22 mm	−1.6 ± 1.2 mm (control group)
		−0.1 ± 1.6 mm (L-PRF group)
	*Horizontal resorption*:	*Horizontal resorption (lingual)*:
	(i) Level A (*control group*): −3.26 ± 0.44 mm	*(Control group)*
	(ii) Level A (*test group*): −2.87 ± 0.25 mm	HW-1 mm: −2.0 ± 2.6 mm
	(iii) Level B (*control group*): −3.82 ± 0.33 mm	HW-3 mm: −0.2 ± 0.3 mm
	(iv) Level B (*test group*): −3.05 ± 0.18 mm	HW-5 mm: −0.1 ± 0.3 mm
		*(Test group)*
		HW-1 mm: −0.3 ± 1.9 mm
		HW-3 mm: −0.1 ± 0.3 mm
		HW-5 mm: −0.0 ± 0.1 mm
	*Mean ISQ test group:*	*Horizontal resorption (buccal)*:
	Immediately after implant placement: 62.33–63.40	*(Control group)*
	1 month postoperatively: 60	HW-1 mm: −3.3 ± 2.6 mm
	3 months postoperatively: 72	HW-3 mm: −1.0 ± 1.1 mm
		HW-5 mm: −0.5 ± 0.7 mm
	*Mean ISQ control group:*	*(Test group)*
	1 month postoperatively:–	HW-1 mm: −1.2 ± 2.6 mm
	3 months postoperatively: 70	HW-3 mm: −0.8 ± 0.9 mm
		HW-5 mm: −0.5 ± 0.6 mm

Secondary outcome	Level B (control group): implants could not be placed in 3 patients	L-PRF group: 94.7% socket fill
		Control group: 63.3% socket fill

**Table 2:** The risk of bias for both studies.

Study	Pang et al. [19]	Temmerman et al. [20]
Random sequence generation	High risk	Low risk
Allocation concealment	High risk	Low risk
Blinding of participants	High risk	Low risk
Blinding of personnel/care providers	High risk	High risk
Detection bias	High risk	High risk
Incomplete outcome data	Low risk	Low risk
Selective reporting	Low risk	Low risk
Group similarity at the baseline	High risk	High risk
Cointerventions	Low risk	Low risk
Compliance	Low risk	Low risk
Intention-to-treat analysis	Low risk	Low risk
Timing of outcome assessments	Low risk	Low risk
Other biases	High risk	High risk
